# Suppression of Protective Responses upon Activation of L-Type Voltage Gated Calcium Channel in Macrophages during *Mycobacterium bovis* BCG Infection

**DOI:** 10.1371/journal.pone.0163845

**Published:** 2016-10-10

**Authors:** Deepika Sharma, Brijendra Kumar Tiwari, Subhash Mehto, Cecil Antony, Gunjan Kak, Yogendra Singh, Krishnamurthy Natarajan

**Affiliations:** 1 Infectious Disease Immunology Lab, Dr. B R Ambedkar Centre for Biomedical Research, University of Delhi, Delhi, India; 2 Department of Zoology, University of Delhi, Delhi, India; Indian Institute of Science, Bangalore, INDIA

## Abstract

The prevalence of *Mycobacterium tuberculosis* (*M*. *tb*) strains eliciting drug resistance has necessitated the need for understanding the complexities of host pathogen interactions. The regulation of calcium homeostasis by Voltage Gated Calcium Channel (VGCCs) upon *M*. *tb* infection has recently assumed importance in this area. We previously showed a suppressor role of VGCC during *M*. *tb* infections and recently reported the mechanisms of its regulation by *M*. *tb*. Here in this report, we further characterize the role of VGCC in mediating defence responses of macrophages during mycobacterial infection. We report that activation of VGCC during infection synergistically downmodulates the generation of oxidative burst (ROS) by macrophages. This attenuation of ROS is regulated in a manner which is dependent on Toll like Receptor (TLR) and also on the route of calcium influx, Protein Kinase C (PKC) and by Mitogen Activation Protein Kinase (MAPK) pathways. VGCC activation during infection increases cell survival and downmodulates autophagy. Concomitantly, pro-inflammatory responses such as IL-12 and IFN-γ secretion and the levels of their receptors on cell surface are inhibited. Finally, the ability of phagosomes to fuse with lysosomes in *M*. *bovis* BCG and *M*. *tb* H37Rv infected macrophages is also compromised when VGCC activation occurs during infection. The results point towards a well-orchestrated strategy adopted by mycobacteria to supress protective responses mounted by the host. This begins with the increase in the surface levels of VGCCs by mycobacteria and their antigens by well-controlled and regulated mechanisms. Subsequent activation of the upregulated VGCC following tweaking of calcium levels by molecular sensors in turn mediates suppressor responses and prepare the macrophages for long term persistent infection.

## Introduction

Tuberculosis, which is caused by *Mycobacterium tuberculosis* (*M*. *tb*) remains as the deadliest infectious disease in the world. Worldwide, in 2014, 9.6 million people were infected with TB; which included 5.4 million men, 3.2 million women and 1 million children. A global estimation has shown that 3.3% of new TB cases and 20% of previously treated cases exhibited Multi Drug Resistance (MDR). It is estimated that of all the MDR-TB cases, a total of 9.7% people display exorbitant resistance against almost known antibiotics against *M*. *tb* and therefore suffer from Extensively Drug Resistance TB (XDR-TB) [1]. Efforts are needed to combat the death toll from the disease in order to achieve the global targets encompassed within Millennium Development Goals (MDGs) [2].

*M*. *tb* modulates pathways like reactive oxygen burst, thereby affecting ROS production [3], apoptosis [4] and autophagy [5] letting it remain undetected within the host. These pathways help in recognising and eliminating the pathogen, thereby clearing off the infection inside the host. In addition, *M*. *tb* thwarts phagosome lysosome fusion [6] and down regulates the expression of MHC class II and Interferon- γ (IFN-γ) on the macrophage surface [7]. Calcium is known to play a crucial role in the *M*. *tb* pathogenesis by differential activation of transcription factors, mediation of phogosome-lysosome fusion and cell survival [8]. Infection by *M*. *tb* is also reported to inhibit calcium mobilization inside macrophages [9].

Calcium response to stimuli consists of two phases [10]. There occurs a rise in calcium concentration, starting with its transient release from the intracellular stores. This event is followed by a more sustained calcium entry across plasma membrane. Calcium Release Activated Channels (CRAC) and Voltage Gated Calcium Channel (VGCC) mediate the second phase of calcium influx [11, 12]. We have previously shown that L-type and R-type calcium channel inhibition in dendritic cells and PBMCs augments the influx of calcium following infection and consequently increases the expression of many pro-inflammatory genes. These genes are known to have a critical role in mediating protective immunity. Knockdown of these genes reduces the bacterial burden drastically within the host cell. We also showed increased expression of VGCCs in the PBMCs of TB patients which were reduced after chemotherapy [13]. We have recently deciphered the mechanisms utilized by this pathogen in regulating VGCC expression in host macrophages. We have shown increased expression of L-type channel upon BCG infection in both human and mouse macrophages. We observed that ROS levels and pCREB were reciprocally regulated which led to altered VGCC expression in macrophages with ROS playing a limiting role towards achieving this [14, 15]. It is known that *M*. *tb* as well as its antigens have the potential to modulate the cytokine milieu in and around the infected cell, further leading to modulation of T cell responses [16–19]. Therefore, keeping in view the role of calcium in *M*. *tb* pathogenesis and the role played by the L-type VGCC, we focussed on the impact of this channel on the protective responses of activated macrophages during *M*. *tb* infection.

Our data indicate that activation of VGCC during infection results in attenuation of ROS production that is dependent upon MyD88 pathway, extra-cellular calcium influx and PKC and MAP-kinase pathways. Further, related defence mechanisms like apoptosis and autophagy were also differentially regulated upon VGCC activation along with infection. This activation also inhibited phagosome-lysosome fusion that was rescued upon its inhibition. We also report downregualtion of IL-12p40 and IFN-γ cytokine and their receptors on macrophages and upregulation of IL-10 cytokine and its receptor during activation of VGCC along with infection.

## Materials and Methods

### Materials

Antibodies against molecules such as Bax, Bcl2, IAP, BECN1, ATG5, MyD88, TRAF6, IRAK1, IRAKM, STIM1, STIM2, ORAI1, GAPDH and siRNAs (both control and specific) to different genes and Luminol Kits for chemiluminescence detection were purchased from Santa Cruz Biotechnologies (Santa Cruz, CA). Chemicals like U0126, 3,4,5-trimethoxybenzoic acid 8-(diethylamino)octyl ester (TMB8), ethylene glycol tetraacetic acid (EGTA), calphostin C and 2’,7’-Dichlorofluorescin diacetate (DCFH-DA) and Fluoroshield with DAPI were purchased from sigma Chemicals Co. (St. Louis MA). L-type calcium channel agonist BAYK8644 [20] and antagonist Amlodipine [21] were purchased from Tocris (United Kingdom). Mouse-Macrophage Colony Stimulating Factor (M-MCSF) and Human-Macrophages Colony Stimulating Factor (H-MCSF) were bought from Invitrogen (US). Lysotracker Red, Lysotracker Green and FM4-64 were purchased from Molecular Probes, Life Technologies (Invitrogen, USA). PE conjugated antibodies to mouse and human IFN-γR (CD119), IL-10R (CD210) and IL-12R (CD212) and the corresponding isotype controls were from BD Biosciences (US). Middlebrook 7H9 (DIFCO) broth was used to grow *M*. *bovis* BCG supplemented with 0.05% Tween 80 and 10% OADC (BD). For all experiments, *M*. *bovis* BCG and H37Rv were used at a Multiplicity of Infection (MOI) of 2. For every infection experiments, bacteria were removed after 4 hours of infection and experimental time point was calculated thereafter.

### Cell culture maintenance and differentiation

In our study, THP-1 monocyte macrophage cell line was used which was a kind gift from Dr. Pawan Malhotra at ICGEB, New Delhi [22]. The RPMI 1640 media supplemented with 2mmol/L L-glutamine and 10% FBS was used to culture cells. THP1 monocytes were differentiated into macrophages via incubation with Phorbol-12-myristate-13-acetate (PMA) with 50ng/ml of PMA for 16 hours. The biopharmacological inhibitors were used against respective molecules; Calphostin C (0.1mM) against PKC, U0126 (10 μM) against MAPK-ERK, TMB-8 (100μM) against IP_3_R and EGTA (3mM) against calcium influx. Incubation of cells was done with the above mentioned reagents for the period of 1 h before BAYK8644 stimulation or infection with *M*. *bovis* BCG or *M*. *tb* H37Rv.

### Ethics Statement for Animal and Human Studies

Every experiment was carried by obtaining approval of the Institutional Animal Ethics and Institutional Human Ethics Committees of Dr. B R Ambedkar Centre for Biomedical Research, University of Delhi, Delhi, India. All the experimental protocols were adhered to the guidelines of the committees. All healthy control/volunteers provided informed written consent to participate in the study; the consent was recorded in English and a vernacular language (Hindi).

### Bone Marrow Derived Macrophages (BMDMs) differentiation

Mouse macrophages were differentiated as described earlier [15]. Briefly, 3–4 weeks old healthy female Balb/c mice were sacrificed and tibias and femur were used as a source of bone marrow. It was flushed out in RPMI 1640 medium. RBCs were lysed using RBC lysis buffer (10X, 12 mM NaHCO_3_, 155 mM NH_4_Cl, 0.1 mM EDTA) for 3 min. B and T lymphocytes and MHC class-II^+^ cells were removed through incubation with CD54R, CD90^+^ and I-A^+^ coupled microbeads respectively followed by Magnet Assisted Cell Sorting (MACS- Miltenyi Biotech, USA) as per the instruction manual. Cells were sorted on the basis of CD11b+ and CDF4/80+ markers. Antibodies used were e Biosciences CD-11b-PE with Catalogue number 11-011-2 and Cd-F4/80- PerCP-Cyanine5.5 with catalogue number 45480180. Cells were suspended in RPMI 1640 medium supplemented with 10% FBS and 40ng/ml Mouse-Macrophage Colony Stimulating Factor (M-CSF) along with sodium pyruvate and 2-mercaptoethanol. Cells were cultured for 5 days in six well culture dishes (non-coated) with fresh medium added on the third day. Infections and stimulations were given after the fifth day.

### Enrichment of human PBMCs and macrophage differentiation

PBMCs were enriched from blood of healthy volunteers as described before [23]. Briefly, PBMCs were isolated from the buffy coat of whole blood over a Histopaque gradient. PBMCs were seeded and allowed to adhere to in a 5% CO_2_ incubator at 37^°^C for 2 hours in the complete media. Non-adherent cells were removed by washing with the media. 0.5-1x10^6^ cells/ml of adherent fraction were cultured in RPMI 1640 containing 50ng/ml human Macrophage-Colony Stimulating Factor (M-CSF), 25 mM HEPES, and 2 mM glutamine and supplemented with 10% autologous human serum for five to seven days, to obtain a homogenous population of differentiated macrophages. Population was sorted on the basis of CD 14+ PE of BD with catalogue number 555398 and biotin CD11b+ with catalogue number 555387. Cytokine addition was done every second day. The cells were either stimulated with *M*. *tb* H37Rv or *M*. *bovis* BCG or FACS analysis was done or western blotted for indicated molecules or processed for confocal microscopy.

### Transfection of THP-1 cells with siRNA

Hiperfect transfection reagent from Qiagen and Optimem medium from Invitrogen was used for transfections as described earlier [24]. Briefly, 1x10^6^/ml PMA differentiated THP-1 macrophages were used in transfection with 60 picomoles of siRNAs against various targeted genes for 36 hours. The knockdown efficiency of the siRNAs was determined by western blotting (S1 Fig. Knockdown efficiency of siRNAs to various molecules). At the end of incubation, cells were stimulated with 50 nM BAYK8644 and/or infected with 2 MOI of *M*. *bovis* BCG and processed for flow cytometry or western blotting as described below.

### Measurement of intracellular Reactive Oxygen Species (ROS)

Flow cytometry was used to measure ROS levels intracellularly in cells cultured in serum free medium and loaded with redox sensitive dye DCFH-DA as described before [24]. DCFH-DA, which is non-fluorescent, readily diffuses into the cells. Once inside the cells, it is hydrolysed to its polar derivative DCFH, which is further oxidized in the presence of H_2_O_2_ to the highly fluorescent DCF. 1X10^6^ cells were incubated with 10 μM DCFH-DA in dark thirty minutes before the end of each incubation period. Cells were washed with PBS and immediately acquired for analysis in FACSCalibur (BD Biosciences). The data was plotted and analysed using CellQuest software. Bars alongside the histograms represent relative Mean Fluorescence intensities of the peak at the higher fluorescence.

### Western blotting for signalling molecules

After respective incubation periods, cells were kept on ice and lysed in buffer containing 0.1M EGTA, 0.1mM EDTA, 10mM KCl, 10mM HEPES (pH 7.9); 0.5% Nonidet P-40 and 2 mg/ml each of aprotinin, leupeptine and pepstatin for about 30 min on ice. This cell suspension was centrifuged at 13,000g for 5 min at 4°C. Supernatant was collected as cytoplasmic extract. Protein estimation was done using Bradford reagent. 25ug of this cytoplasmic protein was resolved on 12% SDS-PAGE and subsequently transferred onto nitrocellulose membrane from Amersham Biosciences, IL. The various molecules of the blots were probed with specific antibodies, followed by HRP-labelled secondary antibody. The luminol reagent was used to develop the blot by chemiluminiscence. Quantification was done using ImageJ software. Parallel gel was western blotted for GAPDH as loading control.

### MTT [3-(4, 5-Dimethylthiozol-2-yl)-2, 5-Dipheyltetrazolium Bromide] Assay

MTT assay was carried out as described recently [23]. Briefly, PMA treated THP-1 cells (1x10^4^ cells/ml), mouse BMDMs and human PBMCs were cultured in a 96 well plate at 37^°^C, infected with 2 MOI of *M*. *bovis* BCG or stimulated with 50nM BAYK8644 or both. After the incubation period cells were washed with PBS. 20uM of MTT solution with concentration of 5mg/ml was added to each well along with 100ul of cell supernatant. Formazan crystals were formed after 4 hours of incubation and those crystals were further dissolved in 100ul of Dimethyl Sulphoxide (DMSO). The absorbance intensity was recorded at 570nm with a reference wavelength of 620nm. All experiments were performed in triplicates.

### Mitochondrial Membrane Potential Assay

Assay for mitochondrial membrane potential was performed as previously described [24]. Briefly, PMA treated THP-1 cells and BMDMs were infected with 2 MOI of *M*. *bovis* BCG and stimulated with 50nM BAYK8644 or both for 24 hours. Cells were stained with 2 μM JC-1 in RPMI-1640 for 30 minutes after the incubation period of various stimulations and infection. Cells were washed with PBS and observed under confocal microscope. JC-1 is carbocynanine dye with a delocalized positive charge. It remains in the monomer form which gives a green fluorescence. Directional uptake of JC-1 monomers in subsequent formation of JC-1 aggregates, which fluoresce at red fluorescence [25], is promoted by the membrane potential of energized mitochondria (negative inside).

### Measurement of Cytokines

Levels of IL-12p40, IL-10 and IFN-γ were estimated in culture supernatants by ELISA, as described previously [24]. Briefly, supernatants of macrophages differentially stimulated/infected were harvested, filtered (0.2 mm filters) and ELISA was performed with Ready-SET-Go ELISA kits from eBioscience (San Diego, USA) strictly as per the manufacturer’s instructions.

### Surface expression of cytokine receptors

Bone marrow derived mouse macrophages and human blood macrophages were stimulated and infected as mentioned above. Cell pellet was collected and washed with PBS. Cells were incubated with specific antibodies to PE-conjugated antibodies to IL-10R, IL-12Rβ and IFN-γR for 30 min at room temperature. Cells were again washed with PBS and immediately acquired on FACS Calibur (BD Biosciences). The data were plotted and analysed using CellQuest Pro software.

### Confocal microscopy for Phagosome-lysosome fusion

PMA activated THP1 cells or mouse macrophages were seeded on the coverslip, washed with RPMI 1640 medium and incubated in OPTIMEM medium with or without BAYK8644 or Amlodipine for 1 h followed by infection with red fluorescent FM4-64 labelled BCG [26] or GFP-expressing *M*. *tb* H37Rv for 4 h. Thirty minutes before the end of infection period, 50 nM of either Lysotracker Green or Lysotracker Red was added to the medium to label lysozyme. After the completion of incubation period, the cells were washed with PBS and fixed with 4% paraformaldehyde for 30 min. cells were washed and mounted with DAPI containing anti-fade. Confocal microscopy was performed on Leica TCS SP-8 confocal instrument, LAS AF Version 2.6.0 build 7266 of Leica Micro Systems CMS GmbH. Images were acquired by sequential scanning to avoid spill over between spectra. The images showing co-localisation were further deconvoluted to show the clear co-localisation between bacterium and lysosomes inside the cell.

### Statistical analysis

To test out the significance of the results, we performed one way ANOVA and followed by Tukey’s post hoc multiple comparison tests. The P value of less than 0.05 was taken as statistically significant difference.

## Results

### Activation of VGCC by BAYK8644 synergistically attenuates oxidative burst

**Fig 1:**
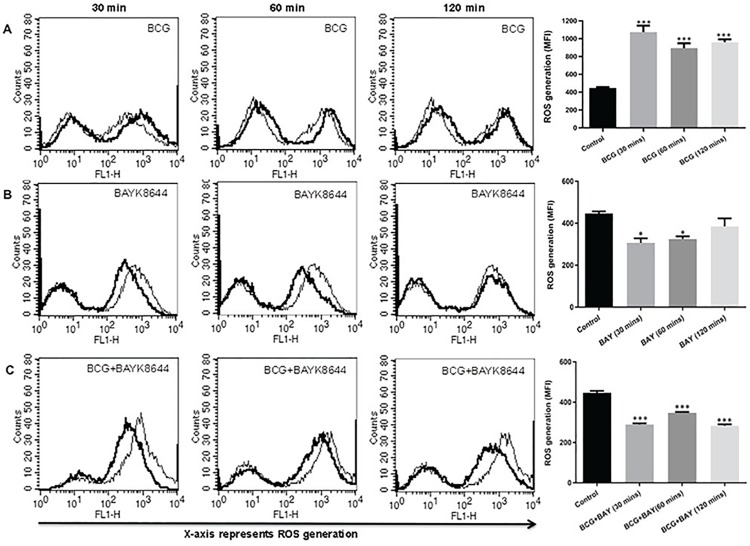
Activation of VGCC along with *M*. *bovis BCG* infection attenuates ROS in THP-1 macrophages. PMA stimulated THP1 macrophages were either infected with 2 MOI *M*. *bo*vis BCG (BCG) or stimulated with 50 nM BAYK8644 or both for indicated times. 30 min before the incubation period, cells were incubated with 10 μM DCFH-DA. After the incubation period, cells were washed with culture medium and ROS levels were analyzed by flow cytometry. In all the Panels, thin line represents ROS generation in uninfected or unstimulated or control cells; thick line represents ROS generation in infected or stimulated cells as indicated. Bar graphs adjacent to histograms in Panel (A-C) show Mean Fluorescent Intensity (MFI) of the peak at the higher fluorescence in the figure. Data from one of three independent experiments are shown (n = 3). The star above the bars represents the P value between control and the corresponding group of that bar in each panel. The results were analyzed by one way ANOVA followed by Tukey’s post hoc multiple comparison test. * = P ≤ 0.05; ** = P ≤ 0.01; *** = P ≤ 0.001 and **** = P ≤ 0.0001.

In order to investigate the role of L-type VGCC activation in modulating key defence mechanisms of macrophages during tuberculosis infection, we stimulated macrophages with L-type calcium channel agonist BAYK8644, a dihydropyridine derivative, which is known to bind to the α1C chain of the channel and regulates calcium entry [20], along with *M*. *bovis* BCG infection and investigated key defence responses. To begin with, we monitored the levels of ROS since our earlier and recent works have pointed towards a regulatory role for ROS in mediating both calcium homeostasis and immune responses [14, 15, 23, 27]. As shown in Fig 1, infection with *M*. *bovis* BCG increased ROS generation at all the indicated time points (panel A). However, stimulation with BAYK8644 significantly decreased ROS levels at all-time points (panel B). Interestingly, costimulation of macrophages with both *M*. *bovis* BCG and BAYK8644 synergistically attenuated ROS production at all-time points (panel C). This indicated a negative role for VGCC in mediating oxidative burst in macrophages during mycobacterial infection. Subsequent experiments were carried out at 1h time point.

**Fig 2:**
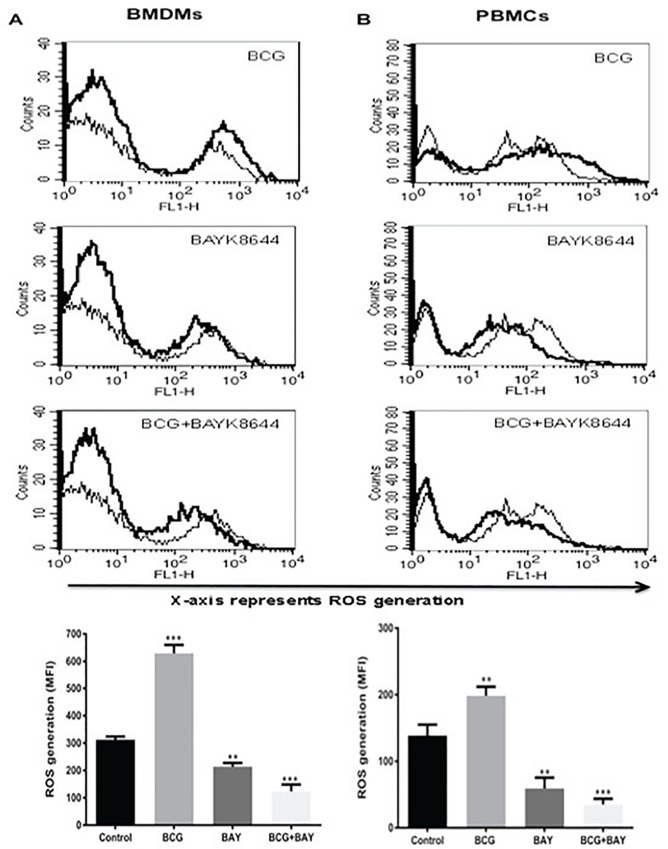
Activation of VGCC along with *M*. *bovis BCG* infection attenuates ROS in mouse and human macrophages. For Panel A, macrophages were derived from bone marrow of female Balb/c mice. For Panel B, PBMCs from healthy volunteers were differentiated into macrophages. Macrophages were either infected with 2 MOI *M*. *bovis* BCG or stimulated with 50 nM BAYK8644 or both for 1h and ROS was estimated as described in Fig 1. In both the Panels, thin line represents ROS generation in uninfected or unstimulated or control cells; thick line represents ROS generation in infected or stimulated cells as indicated. Bar chart below each panel represents MFI of the peak at the higher fluorescence in the figure. Data from one of three independent experiments are shown (n = 3). The star above the bar represents the P value between control and the corresponding group of that bar in each panel. The results were analyzed by one way ANOVA followed by Tukey’s post hoc multiple comparison test. * = P ≤ 0.05; ** = P ≤ 0.01; *** = P ≤ 0.001 and **** = P ≤ 0.0001.

In order to investigate if the above results were not restricted to cell lines, we carried out similar experiments with bone marrow derived mouse macrophages (BMDMs) and human PBMC derived macrophages. As shown in Fig 2, similar results were obtained wherein costimulation with *M*. *bovis* BCG and BAYK8644 synergistically inhibited ROS generation, in both mouse bone marrow macrophages (panel A) as well as human macrophages (panel B). The inhibition was more prominent with human macrophages as compared to mouse macrophages.

### ROS inhibition by VGCC is TLR dependent

**Fig 3:**
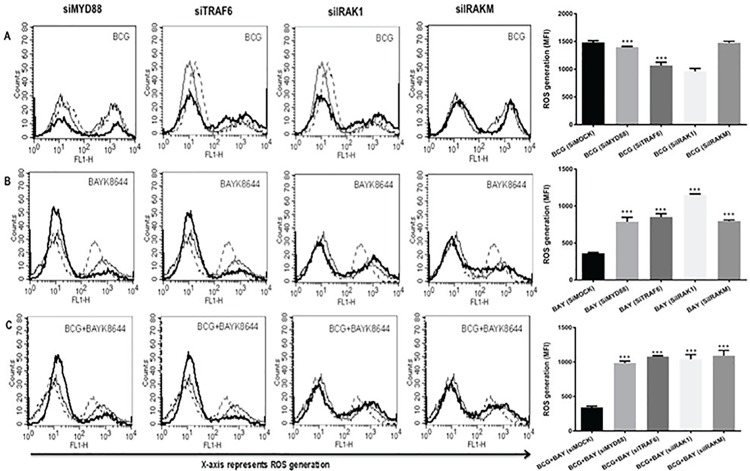
TLR intermediates regulate ROS generation during infection and VGCC activation. PMA simulated THP1 cells were transfected with siRNAs against indicated molecules for 36 h followed by infection with 2 MOI *M*. *bovis* BCG (Panel A) and/or stimulation with 50 nM BAYK8644 (Panel B) or both (Panel C) for 1 h and ROS was estimated as described above. In all the panels, thin line represents ROS generation in unstimulated/uninfected or control cells transfected with control siRNAs. Dotted line represent ROS generation in cells transfected with control siRNA followed by either infection with *M*. *bovis* BCG (Panel A) or stimulation with BAYK8644 (Panel B) or both (Panel C). The thick line in all the panels represents ROS generation in cells either infected with *M*. *bovis* BCG (Panel A) or stimulation with BAYK8644 (Panel B) or both (Panel C) following transfection with specific siRNAs against indicated molecules. Bar graph below each panel represents MFI of the peak at the higher fluorescence in the figure. Data from one of three independent experiments are shown (n = 3). The star above the bar represents the P value between stimulated/infected groups transfected with control siRNAs and the corresponding group of that bar in each panel. The results were analyzed by one way ANOVA followed by Tukey’s post hoc multiple comparison test. ns = P > 0.05; * = P ≤ 0.05; ** = P ≤ 0.01; *** = P ≤ 0.001 and **** = P ≤ 0.0001.

Since TLR signalling is known to play a key part in regulating immunity to pathogens [28, 29], next we probed the role of TLR pathways in regulating ROS production. We individually knockdown the key intermediates in this pathway, namely Myeloid Differentiation Primary Response gene 88 (MyD88), TNF Receptor Associated Factor (TRAF6), Interleukin Receptor Associated Kinase (IRAK)-1 and IRAK-M. S1 Fig represents knockdown efficiency of these intermediates. This was done using specific siRNAs prior to stimulations with BAYK8644 and/or infection with *M*. *bovis* BCG. As shown in Fig 3, knockdown of TRAF6, IRAK1 and to an extent MyD88, but not IRAKM significantly inhibited BCG mediated increased ROS generation (panel A). As IRAKM negatively regulates MyD88 dependent TLR signalling and our results are in agreement with its role [30]. Similarly, knockdown of MyD88, TRAF6, IRAK1 and IRAKM reversed the decrease in ROS production upon VGCC activation and costimulation with *M*. *bovis* BCG and BAYK8644 (panel B and C). These results indicate that inhibition of ROS by VGCC is dependent upon the TLR pathway.

Interestingly, however, stimulation of individual TLRs with their known ligands (TLR2, Pam3Csk4; TLR4, LPS; TLR7, Immiquimoid and TLR9, CpG-DNA) in the presence or absence of *M*. *bovis* BCG infection or stimulation with BAYK8644 or both did not results in any significant modulation of ROS (S2 Fig. Stimulation of TLRs along with VGCC activation and mycobacterial infection has no significant effect on ROS generation). Therefore, our results indicate that TLRs by themselves might not have a significant role in ROS production during BCG infection and/or L-type VGCC activation; however, TLR signalling does seem to regulate ROS production under the above stimulatory conditions.

### Second messengers play a role in the inhibition of ROS by VGCC

**Fig 4:**
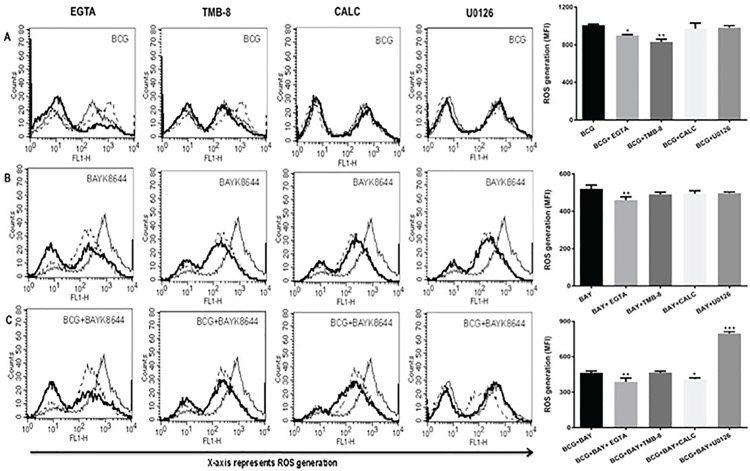
Calcium, MAP-ERK and PKC modulate ROS generation during infection and VGCC activation. PMA stimulated THP1 macrophages were treated with inhibitors to indicated molecules for 1 h followed by either infection with 2 MOI *M*. *bovis* BCG or stimulation with 50 nM BAYK8644 or both for 1 h. In all the panels, thin line represents ROS generation in unstimulated/uninfected or control cells. Dotted line represent ROS generation in cells infected with *M*. *bovis* BCG (Panel A) or stimulation with BAYK8644 (Panel B) or both (Panel C). The thick line in all the panels represent ROS generation in cells either infected with *M*. *bovis* BCG (Panel A) or stimulated with BAYK8644 (Panel B) or both (Panel C) following treatment with inhibitors to indicated molecules. Bar graph below each panel represents MFI of the peak at the higher fluorescence in the figure. Data from one of three independent experiments are shown (n = 3). The star above the bar represents the P value between stimulated/infected cells and the corresponding group of that bar chart in each panel. The results were analyzed by one way ANOVA followed by Tukey’s post hoc multiple comparison test. ns = P > 0.05; * = P ≤ 0.05; ** = P ≤ 0.01; *** = P ≤ 0.001 and **** = P ≤ 0.0001.

To further explore the role of intermediates which are downstream of TLR signalling, biopharmacological inhibitors to key signalling second messengers were used. Extracellular calcium was inhibited using EGTA [31], intracellular calcium release was inhibited with IP**3**R antagonist TMB-8 [32], PKC was inhibited using Calphostin C [33] and ERK-MAP kinase was inhibited using U0126 [34]. As shown in Fig 4, inhibiting calcium from either external influx or intracellular release decreased ROS generation upon BCG infection, while no significant effect was observed upon PKC and MAP Kinase pathway (panel A). This indicated a primary role of calcium for ROS generation during infection. ROS generation upon activation of VGCC on the other hand was regulated only by calcium influx from external medium. Moreover, inhibiting calcium influx further reduced ROS generation upon VGCC activation (panel B). This indicated a differential regulation of ROS by calcium that was dependent upon the nature of the stimulus. Interestingly, ROS generation upon costimulation with *M bovis* BCG and BAYK8644 was further reduced upon inhibiting calcium influx from external medium or PKC (panel C). No apparent role for calcium from internal stores was observed. However, inhibiting ERK-MAPK increased ROS levels upon costimulation that was otherwise unchanged upon individual stimulation. These results point to a unique mechanism of ROS regulation upon VGCC activation during mycobacterial infection.

### Calcium sensing machinery plays a role in ROS inhibition by VGCC

**Fig 5:**
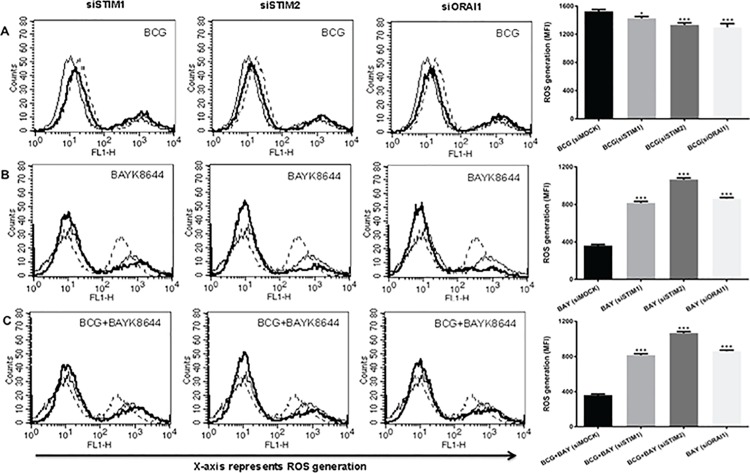
Molecular sensors of calcium influx regulate ROS generation during infection and VGCC activation. PMA stimulated THP-1 cells were transfected with siRNAs against indicated molecules for 36 h followed by infection with 2 MOI *M*. *bovis* BCG and/or stimulation with 50nM for 1 h and ROS was estimated as described above. In all the panels thin line represents ROS generation in unstimulaed/uninfected cells transfected with control siRNAs. Dotted line represent ROS generation in cells transfected with control siRNA followed by either infection with *M*. *bovis* BCG (Panel A) or stimulation with BAYK8644 (Panel B) or both (Panel C). The thick line in all the panels represent ROS generation in cells either infected with *M*. *bovis* BCG (Panel A) or stimulated with BAYK8644 (Panel B) or both (Panel C) following transfection with specific siRNAs against indicated molecules. Bar graph below each panel represents MFI of the peak at higher fluorescence in the figure. Data from one of the three independent experiments are shown (n = 3). The star above the bar represents the P value between unstimulated and the corresponding group of that bar in each panel. The results were analyzed by one way ANOVA followed by Tukey’s post hoc multiple comparison test. * = P< 0.05; ** = P<0.01; *** = P<0.001 and **** = P<0.0001.

We earlier showed the role of intracellular calcium sensing proteins Stromal Interaction Molecule 1 (STIM1) and STIM2, the molecular sensors regulating Store Operated Calcium Entry(SOCE) via Calcium Release Activated Calcium Channel (CRAC) and ORAI1 [11, 35] in regulating calcium homeostasis in the cell and immune responses [23, 36]. We, therefore, dissected their role in governing ROS production during infection and L-type VGCC activation. As shown in Fig 5, knockdown of these genes significantly decreased ROS production upon infection with *M*. *bovis* BCG (panel A), indicating that these sensors positively regulate macrophage ROS production during *M*. *bovis* BCG infection. This complemented the data shown in Fig 4, wherein inhibiting internal calcium stores decreased ROS generation. In contrast, knockdown of these sensors rescued the attenuated ROS levels that were decreased upon VGCC activation (panel B). The same was true when cells were costimulated with *M*. *bovis* BCG and BAYK8644 (panel C). These results aim towards a complex regulation of ROS generation upon infection and VGCC activation. It also indicates to a new strategy adopted by mycobacteria in modulating the roles of molecular sensors of calcium homeostasis to thwart protective responses from macrophages.

### Activation of VGCC promotes macrophage survival and attenuates autophagy

**Fig 6:**
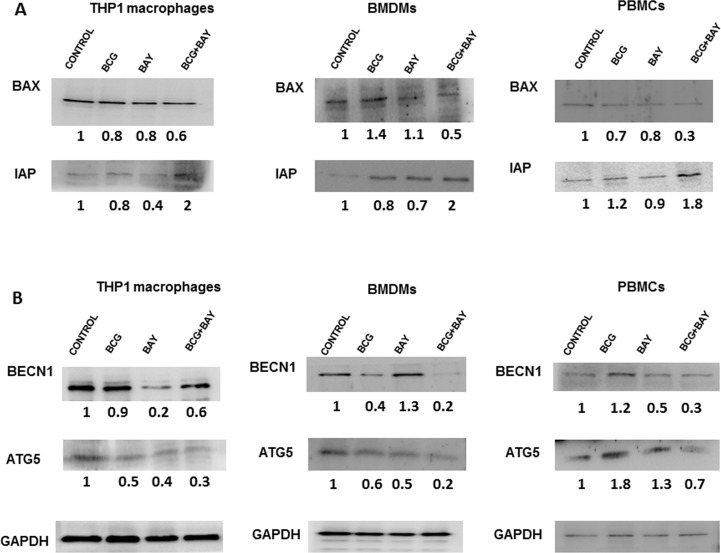
VGCC activation and mycobacterial infection synergistically regulate macrophage survival via apoptosis and autophagy. Either PMA stimulated THP1 macrophages or mouse bone marrow derived macrophages or human PBMC derived macrophages were either infected with 2 MOI *M*. *bovis* BCG or stimulated with 50 nM BAYK8644 or both for 24 h. Cytoplasmic extracts were western blotted for indicated molecules in the apoptotic (Panel A) or autophagic (Panel B) pathways. ‘Control’ represents unstimulated/uninfected cells. Numbers below the blots indicate relative intensities of the bands normalized with the housekeeping molecule GAPDH. All panels show one of three independent experiments (n = 3).

We further explored the role of VGCC in mediating cell survival and autophagy in macrophages during mycobacterial infections. We have recently shown that *M*. *tb* and HIV modulate macrophage apoptosis in a calcium dependent manner [23]. To explore the role of VGCC in this process, THP1 macrophages were stimulated with BAYK8644 along with *M*. *bovis* BCG or virulent *M*. *tb* H37Rv infection. As per Fig 6 panel A, activation of VGCC along with infection with *M*. *bovis* BCG decreased the expression levels of Bax, a pro-apoptotic molecule and there was accompanying increase in the expression level of IAP, an anti-apoptotic molecule upon this co-stimulation. Stimulation with BAYK8644 along with *M*. *bovis* BCG showed similar results in all three cell types (Panel A BMDMs and PBMCs). Similar results were obtained when virulent *M*. *tb* H37Rv was used to infect macrophages along with stimulation with BAYK8644 (S3 Fig. *M*. *tb* H37Rv infection and VGCC activation synergistically regulates apoptosis and autophagy in macrophages). We further monitored survival of cells during VGCC activation and *M*. *bovis* BCG infection. To that end, we monitored cell survival by MTT assay S4 Fig. VGCC activation and mycobacterial infection synergistically regulate macrophage survival, Panel A). Additionally apoptosis was also measured by JC-1 staining. As shown in S4 Fig. Panel B, stimulation of THP-1 macrophages with both BAYK8644 and infection with *M*. *bovis* BCG showed increased red fluorescence in the cells indicative of apoptosis inhibition when compared with individual stimulations. Similar data were obtained for BMDMs. These results indicate a synergistic role of L-type VGCC activation and *M*. *bovis* BCG infection in decreasing apoptosis and prolonging survival of macrophages.

Autophagy is a recently recognized innate defence mechanism that acts as an autonomous system of the cell for eliminating intracellular pathogens and also plays a role in regulating immune responses to pathogens [37]. We have recently shown the role of *M*. *tb* antigens in mediating autophagy in dendritic cells [24]. We moved on to investigate the role of VGCC in mediating autophagy during the infection. For that we monitored the expression levels of autophagy markers BECN1 and ATG5. As shown in Fig 6 Panel B, the expression level of both these autophagy molecules in THP-1, BMDMs as well as human PBMC derived macrophages decreased upon costimulation with BAYK8644 and *M*. *bovis* BCG infection. Similar results were obtained in case of infection with H37Rv (S3 Fig. *M*. *tb* H37Rv infection and VGCC activation synergistically regulates apoptosis and autophagy in macrophages). These results indicate an inhibitory role for VGCC in mediating autophagy during mycobacterial infection.

### Activation of VGCC inhibits phagosome-lysosme fusion *M*. *tb* infection

**Fig 7:**
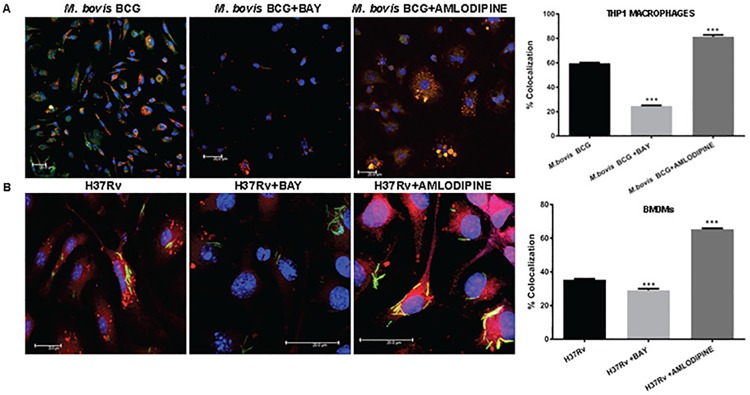
VGCC activation and mycobacterial infection synergistically regulate phagosome-lysosome fusion in macrophages. PMA treated THP1 macrophages (Panel A) and mouse bone marrow derived macrophages (Panel B) were seeded on the coverslip and washed with RPMI 1640 medium and incubated in OPTIMEM medium with or without BAYK8644 for 1 h followed by infection with FM4-64 labeled *M*. *bovis* BCG (panel A) or GFP expressing *M*. *tb* H37Rv (Panel B) for 4 h. thirty minutes prior to the end of infection period, cells were incubated with 50nM of Lysotracker Green (for Panel A) or Lysotracker Green (for Panel B). At the end of incubation period cells were washed once with PBS and fixed with 4% paraformaldehyde for 1h. Following through washes, the cover slips were mounted with anti-fade containing DAPI. Confocal microscopy was performed on Leica TCS SP-8 confocal instrument, LAX Version 1.8.1.137. Bar chart in both Panel A and Panel B represents percentage of co-localization as determined by LAS AF Version 2.6.0 build 7266 of Leica Micro Systems CMS GmbH. Bars represent percentage of co-localization of the indicated groups of three independent experiments (n = 3). The stars represent the P value between unstimulated and corresponding stimulated (Bay/Amlodipine) group of that bar in each panel. The results were analyzed by one way ANOVA followed by Tukey’s post hoc multiple comparison test. * = P< 0.05; ** = P<0.01; *** = P<0.001 and **** = P<0.0001.

A major defence mechanism of *M*. *tb* in macrophages is the prevention of fusion between phagosomes and lysosomes that allows the pathogen to establish long-term persistent infections [37]. It has also been demonstrated that phagosome-lysosome fusion is mediated by calcium homeostasis [38]. Keeping this in mind, we investigated the role of VGCC in mediating phogosome-lysosome fusion. We used FM4-64 labelled *M*. *bovis* BCG (red) to infect THP-1 macrophages and the fusion of bacteria to the lysosome (labelled with Lysotracker Green) were monitored during both VGCC activation with BAYK8644 and VGCC inhibition with Amlodipine [21]. We similarly monitored phagosome-lysosome fusion in GFP-expressing *M*. *tb* H37Rv (green) infected and BAYK8644 and Amlodipine stimulated macrophages labelled with lysotracker red. As shown in Fig 7, costimulation of THP1 macrophages (panel A) with *M*. *bovis* BCG or costimulation of BMDMs with *M*. *tb* H37Rv (panel B) infection along with BAYK8644 further reduced phagosome-lysosome fusion. In contrast, inhibiting VGCC with amlodipine now boosted phagosome-lysosome fusion. These results clearly indicate that activation of VGCC during mycobacterial infection further aids the pathogen to establish persistent infection inside macrophages.

### Activation of VGCC during infection leads to suppression of pro-inflammatory responses and activation of anti-inflammatory responses

**Fig 8:**
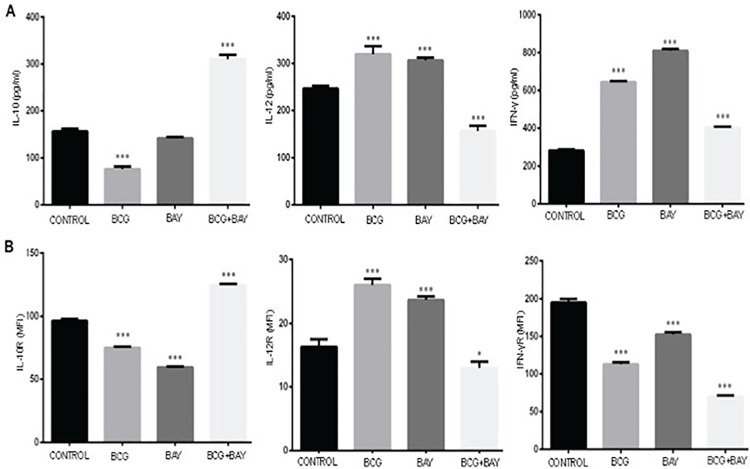
VGCC activation and mycobacterial infection synergistically lead to suppression of pro-inflammatory cytokine responses in mouse macrophages. Bone marrow derived mouse macrophages (Panel A&B) either were infected with 2 MOI of *M*. *bovis* BCG or stimulated with 50nM of BAYK8644 or both for 24 h. For Panel A, culture supernatant was processed for the estimation of indicated cytokines and bars represent the amount of cytokine in pg/ml. For Panel B, cells were processed for measuring surface densities of indicated cytokine receptors by FACS and bars represent MFI of indicated groups of three independent experiments (n = 3). The results were analyzed by one way ANOVA followed by Tukey’s post hoc multiple comparison test. The star above the bar represents the P value between unstimulated/uninfected or control and the corresponding group of that bar in each panel. ns = P>0.05; * = P< 0.05; ** = P<0.01; *** = P<0.001 and **** = P<0.0001.

**Fig 9:**
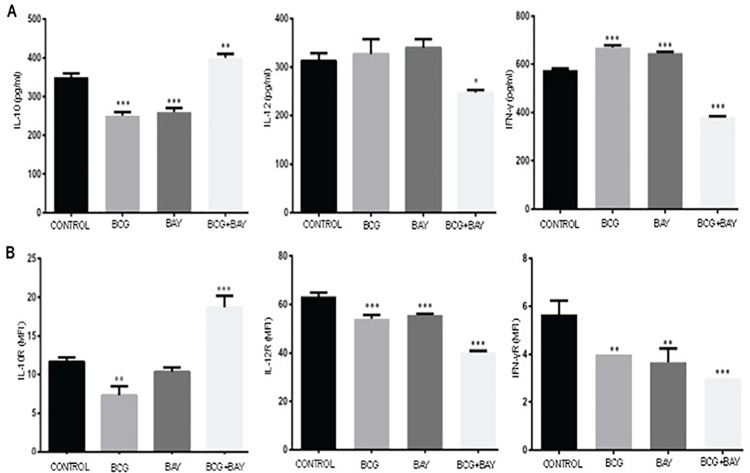
VGCC activation and mycobacterial infection synergistically lead to suppression of pro-inflammatory cytokine responses in human macrophages. Human PBMC derived macrophages (Panel A&B) either were infected with 2 MOI *M*. *bovis* BCG or stimulated with 50 nM BAYK8644 or both for 24 h. For Panel A, culture supernatant was processed for the estimation of indicated cytokines and bars represent the amount of cytokine in pg/ml. For Panel B, cells were processed for measuring surface densities of indicated cytokine receptors by FACS and bars represent MFI of indicated groups of three independent experiments (n = 3). The results were analyzed by one way ANOVA followed by Tukey’s post hoc multiple comparison test. The star above the bar represents the P value between unstimulated/uninfected and the corresponding group of that bar in each panel. ns = P>0.05; * = P< 0.05; ** = P<0.01; *** = P<0.001 and **** = P<0.0001.

A differential cytokine profile is known to affect the outcome of *M*. *tb* infection. They impart elemental effects on further developing T cell responses. The relative densities of cytokine receptors on macrophage surface are also responsible for the bifurcation of the immune responses into Th1 and Th2. We therefore, investigated the profile of key cytokines as well as their receptors on macrophage surfaces during infection and VGCC activation. Since primary cells are better producers of cytokines as compared to cell lines, we restricted the study to mouse BMDMs and PBMC derived human macrophages. As shown in Figs 8 and 9, the costimulation resulted in an increase in the levels of IL-10 and a significant decrease in the levels of IL-12 and IFN-γ (Figs 8A and 9A). Concomitantly, costimulation with BAYK8644 along with *M*. *bovis* BCG infection, decreased the surface levels of IFN-γ and IL-12 receptors and increased the levels of IL-10 receptor in both mouse (Fig 8B) as well human macrophages (Fig 9B). The decrease in IFN-γ was more prominent in case of human PBMCs. These results once again point towards a suppressor effect of VGCC activation during mycobacterial stimulation.

## Discussion

*M*. *tb* has developed multiple strategies to escape the protective immune responses by modulating the host factors [6, 39]. With the emergence of drug resistance, it is imperative to devise strategies that effectively control and eventually eradicate TB infection. Keeping this in mind, our lab has been investigating the intricacies of host-pathogen interactions at the cellular and molecular levels. We earlier showed that many *M*. *tb* antigens induce the differentiation and activation of dendritic cells. The antigen differentiated DCs induced suppressor responses to *M*. *tb*. This included downmodulation of Th1 responses [40], attenuation of pro-inflammatory cytokines and chemokines [16] and the production of ROS [27]. Elucidation of the pathways mediating these responses identified calcium homeostasis as a master regulator [41].

Calcium has been known to play regulatory roles in many of the immune evasion mechanisms targeted by *M*. *tb*. Further, it has been reported that activities of Calmodulin, Sphingosine Kinase and PKC are regulated by calcium, affecting the survival of *M*. *tb* [9]; and Calcineurin which in turn regulates the phagosomal expression of coronin-1 eventually affecting the maturation of phagosome [42]. The cascading effect is produced through downstream kinase activation via MAPK pathway. This further regulates phosphorylation status of transcription factors like c-myc and CREB. Building on this, we recently explained the mechanism employed by *M*. *tb* and its antigens for regulating the expression profile of VGCC on macrophage surface. We observed a crucial role for ROS and the transcription factor pCREB [14, 15]. We have recently observed similar roles for calcium in other infections [23, 43]. We identified VGCC as a negative regulator of calcium influx in DCs and macrophages. Infected and antigen activated DCs, macrophages and PBMCs of TB patients displayed increased levels of L-type VGCC on their surface. VGCC blockade induced protective responses at the cellular and intracellular levels and attenuated bacterial loads in mice [13]. These results were later also observed by Ramakrishnan and co-workers [44] and Bishai and co-workers [45]. VGCC has also emerged as a key regulator of calcium induction in CD4^+^ T cells during *Leishmania* infections [46].

As a next plausible step in this direction, we characterized the roles that VGCC might play in modulating host protective immune responses. To that end we employed a known activator of VGCC namely, BAYK8644, along with *M*. *bovis* BCG and *M*. *tb* H37Rv infections. Key responses that are makers for protection were investigated.

Macrophages are known to increase the production of ROS upon infection as it has bactericidal activity [47]. This is in agreement with our data wherein *M*. *bovis* BCG infection led to increased ROS production. This increased ROS production could be the result of various cytokines upregulated or downregulated during infection. ROS in itself is important to further influence processes like apoptosis and autophagy in the infected macrophages. Calcium and ROS have been known to affect each other [48]. We here show that increased calcium influx upon stimulation with BAYK8644 leads to decrease in ROS production. Costimulation of VGCC along with infection led to synergistic inhibition of ROS production. This suggests dominant role for calcium and channel activation in regulating ROS production by macrophages. We propose that the bacterium would upregulate the expression of VGCC and increase calcium influx thereby decreasing ROS production and allowing its survival inside the host macrophages.

Pathogens are known to hijack the TLR signalling to evade the recognition and elimination by immune system because TLR2 mediated signals preferentially induces Th2 kind of immune response [49]. Others like *Yersinia enterocolitica* and *Candida albicans* exploit TLR2 dependent IL-10 release leading to immunosuppression [50]. In this study, we show that increased ROS production by macrophages is TLR dependent and acts in a MyD88 independent pathway involving IRAK1 and TRAF6. This shows that calcium heavily interferes with the TLR signalling during mycobacterial infection. Expectedly, when channel is activated along with infection we see a similar profile of ROS dependency upon TLR intermediates. Interestingly, the ERK-MAPK pathway had a differential role to play; while it had minimal role in individual stimulations, inhibiting ERK during costimulations reversed the attenuation of ROS. This implies that cellular signalling is altered during infection along with channel activation. Interestingly, calcium homeostasis played a different and contrasting roles during VGCC attenuated ROS production. Calcium influx from external source played a positive role only during ROS production during *M*. *bovis* BCG infection. Similarly, molecular sensors of calcium also mediated fine and differential regulation of ROS production. While they played positive roles in ROS production during mycobacterial infection, they had a negative role in ROS production during VGCC activation as well as during costimulation of VGCC along with *M*. *bovis* BCG infection. These results point to a dominant effect of VGCC activation over *M*. *bovis* BCG infection and indicate that signalling mechanisms are tweaked by VGCC to inhibit ROS production. We are in the process of identifying these mechanisms and initial results point towards differential association of kinases with the cytoplasmic tails of VGCC that might play a role in this process (data not shown).

We next investigated the distal effects of VGCC activation. Our results indicate that VGCC activation inhibit apoptosis and autophagy, key defence mechanisms of macrophages that are targeted by *M*. *tb*. The release of intracellular component is prevented by apoptosis thereby limiting the spread of infection. There are evidences suggesting that the apoptotic response of macrophages to mycobacterial infections play a role in defence against these microorganisms [51]. Classical apoptosis has been known to promote host defences in TB by denying infecting bacterium a protected intracellular environment for its replication [52]. It is known that the apoptotic cell death of *M*. *tb* infected macrophages is associated with mycobacterial killing [53] and is also known to increase the T-cell responses by detouring the pathway of antigen presentation [54]. *M*. *tb* prevents programmed cell death of infected macrophages which reduces antigen presentation [55]. We recently reported a synergistic inhibition of apoptosis during *M*. *tb*-HIV co-infection [23]. This also employed calcium homeostasis and TLR intermediates. Concurrent with these observations, the results in this study show that the L-type VGCC activation along with infection reduces apoptosis of macrophages. It has been reported that apoptosis effectively controls bacterial growth at early time points and can contribute in generation of antigen specific CD8 T cells at later stages.

We also highlight an inhibitory role of L-type VGCC activation in mediating autophagy during BCG infection. Autophagy is also known to facilitate the trafficking of bacterium to lysosomes leading to degradation [56]. Inhibition of autophagy therefore will inhibit elimination of the intracellular pathogen as autophagy is known to play a role in innate defence mechanism. Animal models have proven that defective autophagy leads to increased bacterial burden in lungs of *M*. *tb* challenged animals [57].

Similarly, we also showed that VGCC activation leads to inhibition of phagosome-lysosome fusion, the target of *M*. *tb* that leads to the establishment of persistent infections. Studies demonstrate that failure of mycobacterial phagosome to mature into acidic, microbicidal phagosome and inhibition of macrophage calcium signalling are tightly coupled [9]. We propose a mechanism wherein bacteria upregulate VGCC, thereby inhibiting calcium signalling and causing decreased phagosome-lysosome fusion.

Finally, a suppressor role for VGCC was also observed in regulating cytokine and their receptor levels on macrophage surface. Th1 responses are dependent upon the increase in levels of IL-12p40 and IFN-γ in antigen presenting cells. And increase in the levels of IL-10 and/or TGF-β leads to Th2 or regulatory responses [16]. It has been shown that mycobacteria and their antigens downmodulate IFN-γ receptor on macrophage surface [7]. We also demonstrated a similar role for many *M*. *tb* antigens in modulating cytokines and cytokine receptors on dendritic cells [27]. Our results show that VGCC down-modulated the levels of pro-inflammatory and protective cytokines and their receptors while increasing the levels of suppressor cytokine IL-10. Our results point to a clear inhibitory role for VGCC during mycobacterial infection. Many of these cytokines would eventually be responsible for processes like apoptosis and autophagy.

As mentioned earlier, much work has been done on efflux pump inhibitors, particularly L-type VGCC inhibitor verapamil, as an additive to already existing TB drug regimen [44, 45]. It has been speculated that the L-type channel blockage by verapamil impacts the intracellular calcium signalling thereby strengthening the immunity of immune cells [58]. Collectively these results point to a unique strategy adopted by *M*. *tb* to subvert the host defence mechanisms. This begins with the expression of proteins/antigens that enhance the cell surface expression of VGCC. The enhanced expression and subsequent activation of VGCC shifts the otherwise protective responses into immune suppressor responses leading to the establishment of persistence expression.

## Supporting Information

S1 Fig Knockdown efficiency of siRNAs to various molecules. PMA stimulated THP1 cells were transfected with siRNAs to indicated molecules for 36h. Cytoplasmic extracts were prepared and western blotted for indicated molecules. MOCK represents cells transfected with control siRNAs. Numbers below the blots indicate relative intensities of the bands normalized with the housekeeping molecule GAPDH
(DOCX)

S2 Fig Stimulation of TLRs along with VGCC activation and mycobacterial infection has no significant effect on ROS generation. For Panel A, PMA stimulated THP1 macrophages were stimulated with known ligands of TLRs for 1 h and ROS production was monitored as described in Fig 1. For Panel B PMA stimulated THP1 macrophages were stimulated with known ligands of TLRs for one hour followed by either infection with 2 MOI *M*. *bovis* BCG or stimulation with 50 nM BAYK8644 or both and ROS was estimated as in described in Fig 1. For Panel A, thin line represents ROS generation by unstimulated cells. Thick line represents ROS generation by cells stimulated with indicated ligands to different TLRs. For Panel B, the thin line represents ROS generation by unstimulated or uninfected cells; dotted line represents ROS generation by cells either infected with 2 MOI *M*. *bovis* BCG or stimulated with 50 nM BAYK8644 or both. The thick line represents ROS generation by cells either stimulated with 50 nM BAYK8644 or infected with 2 MOI *M*. *bovis* BCG along with stimulations with indicated ligands to different TLRs.
(DOCX)

S3 Fig *M*. *tb* H37Rv infection and VGCC activation synergistically regulates apoptosis and autophagy in macrophages. PMA stimulated THP1 macrophages were either infected with 2 MOI *M*. *tb* H37Rv or stimulated with 50 nM BAYK8644 or both for 24 h. Cytoplasmic extracts were western blotted for indicated molecules. ‘Control’ represents uninfected cells. Numbers below the blots indicate relative intensities of the bands normalized with GAPDH. All panels show one of three independent experiments.
(DOCX)

S4 Fig VGCC activation and mycobacterial infection synergistically regulate macrophage survival. For Panel A, PMA stimulated THP1 macrophages or mouse bone marrow derived macrophages (BMDMs) or human PBMC derived macrophages were either infected with 2 MOI *M*. *bovis* BCG (BCG) or stimulated with 50 nM BAYK8644 (BAY) or both for 24 h. Cell survival as represented by percent viability was determined by MTT assay. Data from one of three independent experiments are shown (n = 3). The star above the bars represents the P value between that group and uninfected or unstimulated or control group in each panel. The results were analyzed by one way ANOVA followed by Tukey’s post hoc multiple comparison test. * = P ≤ 0.05; ** = P ≤ 0.01; *** = P ≤ 0.001 and **** = P ≤ 0.0001. For Panel B, PMA stimulated THP1 macrophages or mouse bone marrow derived macrophages were either infected with 2 MOI M. bovis BCG or stimulated with 50 nM BAYK8644 or both for 24 h. Cells were stained with JC-1 dye for 30 mins and observed under Nikon C2 confocal microscope.
(DOCX)
