# Supplementary material for: Suppression of Protective Responses upon Activation of L-Type Voltage Gated Calcium Channel in Macrophages during Mycobacterium bovis BCG Infection
Source: PLoS One. 2016 Oct 10;11(10):e0163845. doi: 10.1371/journal.pone.0163845 (PMC5056721; doi:10.1371/journal.pone.0163845)
Supplement: S1 Fig — PMA stimulated THP1 cells were transfected with siRNAs to indicated molecules for 36h. Cytoplasmic extracts were prepared and western blotted for indicated molecules. MOCK represents cells transfected with control siRNAs. Numbers below the blots indicate relative intensities of the bands normalized with the housekeeping molecule GAPDH (DOCX) [file pone.0163845.s001.docx]

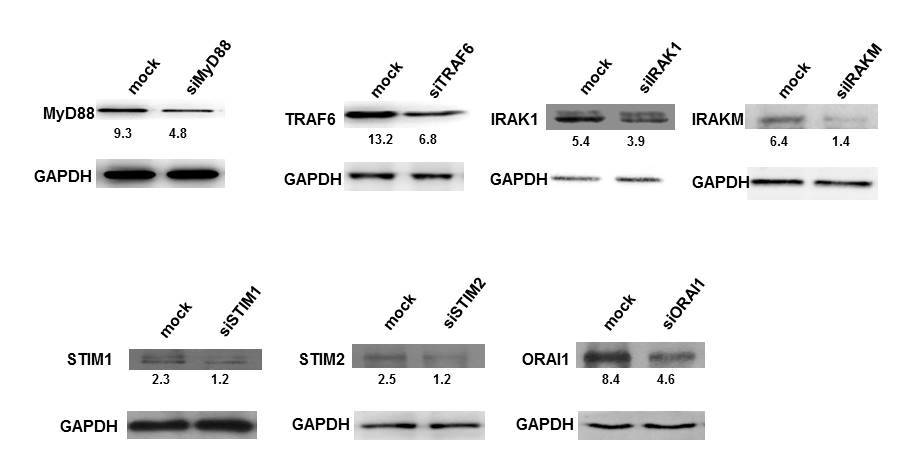


**Figure S1. Knockdown efficiency of siRNAs to various molecules.** PMA stimulated THP1 cells were transfected with siRNAs to indicated molecules for 36h. Cytoplasmic extracts were prepared and western blotted for indicated molecules. MOCK represents cells transfected with control siRNAs. Numbers below the blots indicate relative intensities of the bands normalized with the housekeeping molecule GAPDH.
