# Supplementary material for: Suppression of Protective Responses upon Activation of L-Type Voltage Gated Calcium Channel in Macrophages during Mycobacterium bovis BCG Infection
Source: PLoS One. 2016 Oct 10;11(10):e0163845. doi: 10.1371/journal.pone.0163845 (PMC5056721; doi:10.1371/journal.pone.0163845)
Supplement: S2 Fig — For Panel A, PMA stimulated THP1 macrophages were stimulated with known ligands of TLRs for 1 h and ROS production was monitored as described in Fig 1. For Panel B PMA stimulated THP1 macrophages were stimulated with known ligands of TLRs for one hour followed by either infection with 2 MOI M. bovis BCG or stimulation with 50 nM BAYK8644 or both and ROS was estimated as in described in Fig 1. For Panel A, thin line represents ROS generation by unstimulated cells. Thick line represents ROS generation by cells stimulated with indicated ligands to different TLRs. For Panel B, the thin line represents ROS generation by unstimulated or uninfected cells; dotted line represents ROS generation by cells either infected with 2 MOI M. bovis BCG or stimulated with 50 nM BAYK8644 or both. The thick line represents ROS generation by cells either stimulated with 50 nM BAYK8644 or infected with 2 MOI M. bovis BCG along with stimulations with indicated ligands to different TLRs. (DOCX) [file pone.0163845.s002.docx]

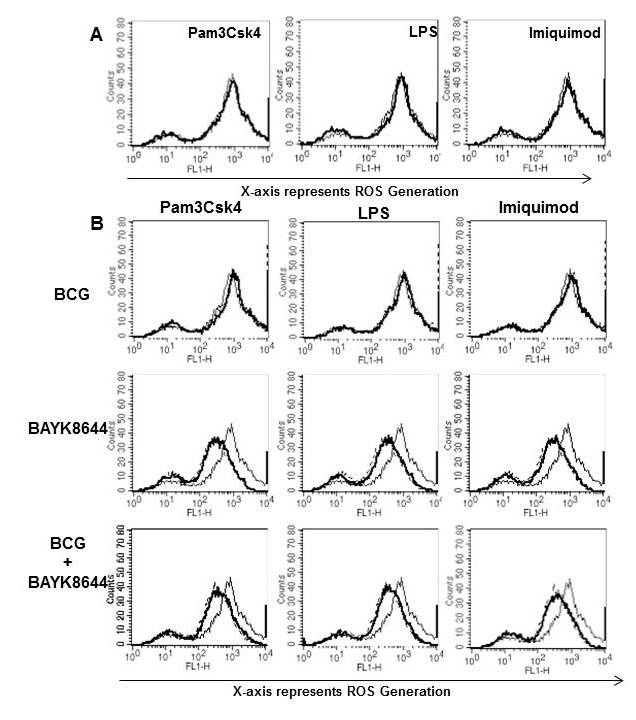


**Figure S2. Stimulation of TLRs along with VGCC activation and mycobacterial infection has no significant effect on ROS generation.** For Panel A, PMA stimulated THP1 macrophages were stimulated with known ligands of TLRs for 1 h and ROS production was monitored as described in Figure 1. For Panel B PMA stimulated THP1 macrophages were stimulated with known ligands of TLRs for one hour followed by either infection with 2 MOI *M. bovis* BCG or stimulation with 50 nM BAYK8644 or both and ROS was estimated as in described in Figure 1. For Panel A, thin line represents ROS generation by unstimulated cells. Thick line represents ROS generation by cells stimulated with indicated ligands to different TLRs. For Panel B, the thin line represents ROS generation by unstimulated or uninfected cells; dotted line represents ROS generation by cells either infected with 2 MOI *M. bovis* BCG or stimulated with 50 nM BAYK8644 or both. The thick line represents ROS generation by cells either stimulated with 50 nM BAYK8644 or infected with 2 MOI *M. bovis* BCG along with stimulations with indicated ligands to different TLRs.
