# Supplementary material for: Suppression of Protective Responses upon Activation of L-Type Voltage Gated Calcium Channel in Macrophages during Mycobacterium bovis BCG Infection
Source: PLoS One. 2016 Oct 10;11(10):e0163845. doi: 10.1371/journal.pone.0163845 (PMC5056721; doi:10.1371/journal.pone.0163845)
Supplement: S3 Fig — PMA stimulated THP1 macrophages were either infected with 2 MOI M. tb H37Rv or stimulated with 50 nM BAYK8644 or both for 24 h. Cytoplasmic extracts were western blotted for indicated molecules. ‘Control’ represents uninfected cells. Numbers below the blots indicate relative intensities of the bands normalized with GAPDH. All panels show one of three independent experiments. (DOCX) [file pone.0163845.s003.docx]

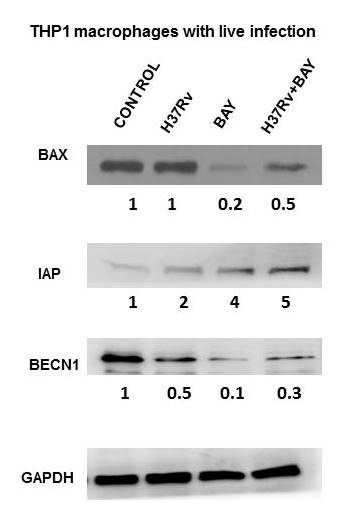


**Figure S3. *M. tb* H37Rv infection and VGCC activation synergistically regulates apoptosis and autophagy in macrophages.** PMA stimulated THP1 macrophages were either infected with 2 MOI *M. tb* H37Rv or stimulated with 50 nM BAYK8644 or both for 24 h. Cytoplasmic extracts were western blotted for indicated molecules. ‘Control’ represents uninfected cells. Numbers below the blots indicate relative intensities of the bands normalized with GAPDH. All panels show one of three independent experiments.
