# Supplementary material for: Suppression of Protective Responses upon Activation of L-Type Voltage Gated Calcium Channel in Macrophages during Mycobacterium bovis BCG Infection
Source: PLoS One. 2016 Oct 10;11(10):e0163845. doi: 10.1371/journal.pone.0163845 (PMC5056721; doi:10.1371/journal.pone.0163845)
Supplement: S4 Fig — For Panel A, PMA stimulated THP1 macrophages or mouse bone marrow derived macrophages (BMDMs) or human PBMC derived macrophages were either infected with 2 MOI M. bovis BCG (BCG) or stimulated with 50 nM BAYK8644 (BAY) or both for 24 h. Cell survival as represented by percent viability was determined by MTT assay. Data from one of three independent experiments are shown (n = 3). The star above the bars represents the P value between that group and uninfected or unstimulated or control group in each panel. The results were analyzed by one way ANOVA followed by Tukey’s post hoc multiple comparison test. * = P ≤ 0.05; ** = P ≤ 0.01; *** = P ≤ 0.001 and **** = P ≤ 0.0001. For Panel B, PMA stimulated THP1 macrophages or mouse bone marrow derived macrophages were either infected with 2 MOI M. bovis BCG or stimulated with 50 nM BAYK8644 or both for 24 h. Cells were stained with JC-1 dye for 30 mins and observed under Nikon C2 confocal microscope. (DOCX) [file pone.0163845.s004.docx]

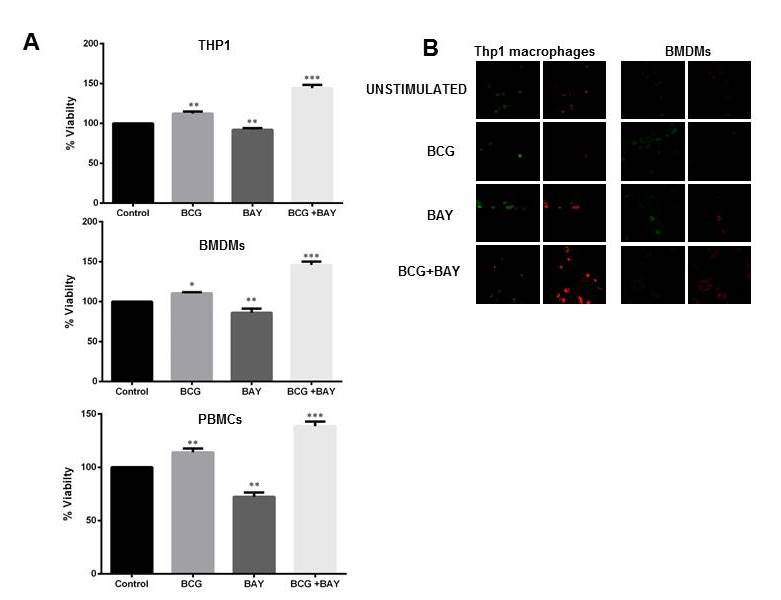


**Figure S4. VGCC activation and mycobacterial infection synergistically regulate macrophage survival.** For Panel A, PMA stimulated THP1 macrophages or mouse bone marrow derived macrophages (BMDMs) or human PBMC derived macrophages were either infected with 2 MOI *M*. *bovis* BCG (BCG) or stimulated with 50 nM BAYK8644 (BAY) or both for 24 h. Cell survival as represented by percent viability was determined by MTT assay. Data from one of three independent experiments are shown (n=3). The star above the bars represents the P value between that group and uninfected or unstimulated or control group in each panel. The results were analyzed by one way Anova followed by Tukey’s post hoc multiple comparison test. *=P ≤ 0.05; **=P ≤ 0.01; ***=P ≤ 0.001 and **** =P ≤ 0.0001. For Panel B, PMA stimulated THP1 macrophages or mouse bone marrow derived macrophages were either infected with 2 MOI *M. bovis* BCG or stimulated with 50 nM BAYK8644 or both for 24 h. Cells were stained with JC-1 dye for 30 mins and observed under Nikon C2 confocal microscope.
